# Cationic Gold on Heteroatom Doped Carbon Supports for Vinyl Chloride Production

**DOI:** 10.1007/s10562-026-05351-2

**Published:** 2026-03-18

**Authors:** Joseph Cartwright, Hannaneh Hosseini, Alexander Gunnarson, Anna Lazaridou, Jonathan M. Mauß, Ben Davies, Samuel Pattisson, Angeles Lopez-Martin, David J. Morgan, Nicholas F. Dummer, Ferdi Schüth, Graham J. Hutchings

**Affiliations:** 1 Cardiff Catalysis Institute, School of Chemistry, Max Planck-Cardiff Centre on the Fundamentals of Heterogeneous Catalysis FUNCAT, Cardiff University, Translational Research Hub, Cardiff, CF24 4HQ UK; 2 Department of Heterogeneous Catalysis, Max-Planck-Institut für Kohlenforschung, Kaiser-Wilhelm-Platz 1, 45470 Mülheim an der Ruhr, Germany

**Keywords:** Vinyl chloride monomer, Au, Heteroatom, Dopant, Activated carbon, Heterogeneous catalysis

## Abstract

Replacement of mercuric chloride catalysts to produce vinyl chloride monomer from acetylene, the precursor to PVC, is needed due to widespread environmental damage of leached mercury. Cationic gold catalysts, which have been recently commercialised, represent a more environmentally benign alternative. However, new catalysts are required to limit the atomically dispersed cationic Au from agglomeration due to reduction under reaction conditions. Several strategies are available to stabilise the Au active sites such as the use of sulphur containing ligands or to use heteroatom doped carbon as the support. Here we prepared two types of doped carbon supports; spheres derived via a hard template methodology and secondly, doped commercial activated carbon. In both cases the Au supported on S-doped carbon was superior in comparison to the undoped analogue; the acetylene conversion was enhanced by 1.6 times over the S-doped carbon sphere catalyst and 2 times over the S-doped Norit catalyst at 60 min time-on-line. The stability and activity of the gold centres are discussed with respect to the heteroatom used, in this case either sulphur, nitrogen, or a combination of the two, and compared to the unmodified supports.

**Graphical Abstract:**

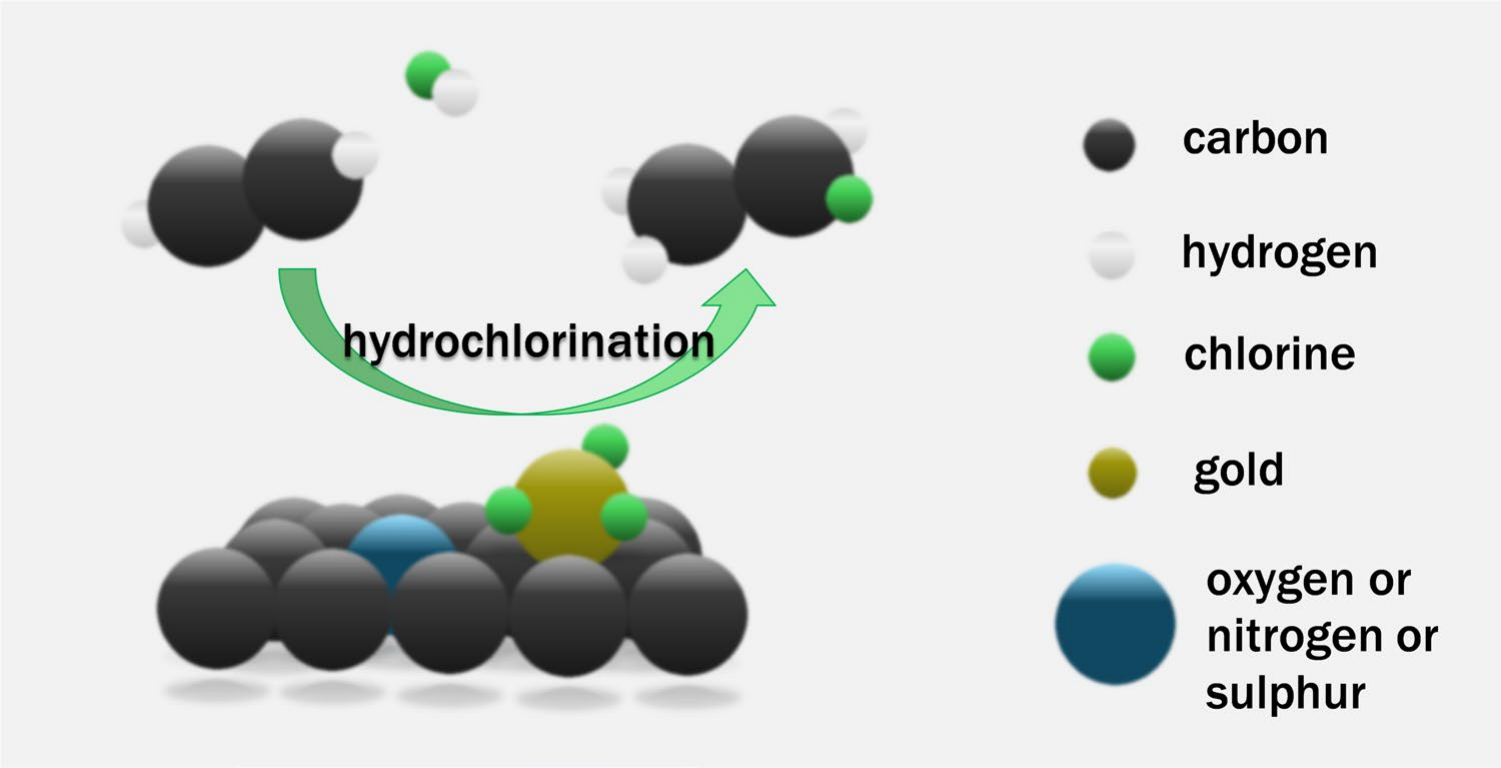

**Supplementary Information:**

The online version contains supplementary material available at 10.1007/s10562-026-05351-2.

## Introduction

Vinyl chloride monomer (VCM) is a critical building block in the petrochemical industry, with an estimated global market value of 91 Bn USD and production volume of 6.7 Mt in 2024 [1]. The majority of VCM is used in the production of polyvinyl chloride (PVC), a highly durable and versatile thermoplastic, which is employed across various industries, including construction, packaging, electronics and apparel [1, 2]. The increased demand for PVC in the construction sector and automotive sectors has driven a significant growth in demand, with a predicted compound annual growth rate (CAGR) of 7.4% until 2034 [1].

VCM can be produced via two routes: ethylene oxychlorination to ethylene dichloride (EDC), followed by thermal dehydrochlorination, or the direct hydrochlorination of acetylene. While the ethylene-based process has a lower overall yield (approximately 50% per pass) and requires harsh reaction conditions, the direct hydrochlorination of acetylene achieves nearly full conversion rates with high selectivity at moderate temperatures [3–5]. However, two-thirds of global VCM production are carried out via the ethylene-based process, due to the use of toxic mercury catalysts (HgCl_2_/C) for the hydrochlorination of acetylene. The Hg catalyst is volatile under reaction conditions, leading to emissions of up to 0.6 kg of Hg per ton of VCM produced [2, 5–7]. A non-toxic alternative is urgently needed, particularly as acetylene can be derived from sustainable sources, such as (bio)methane from biomass fermentation or CO_2_ methanation via an electric non-thermal plasma pyrolysis process, allowing for the synthesis of renewable VCM [7–11].

Over the past decades, various noble metal (e.g. Ag^+^, Pd^2+^, Rh^3+^), non-noble metal (e.g. Cu^2+^, Bi^3+^, Sn^2+^) and non-metal (e.g. N-doped carbon) catalyst alternatives have been investigated [2, 3, 5, 8]. As pioneered by Hutchings and coworkers, atomically dispersed Au^3+^ on activated carbon (AuCl_3_/C) is widely recognized as the best drop-in solution for Hg catalysts due to its superior catalytic activity [2, 3, 8]. However, Au catalysts are prone to rapid deactivation on stream, which is attributed to the formation of carbonaceous deposits at low temperatures (< 100 °C) and the reduction of cationic Au species (Au^3+^/Au^+^) to metallic Au (Au^0^) that agglomerates to nanoparticles at higher temperatures (> 120 °C) [3, 5–7]. Metal additives (e.g. Cu, Ce, Co) can increase the activity and stability of Au catalysts by tailoring adsorptive properties towards HCl and C_2_H_2_ and donating electron density to cationic Au species. However, the prospect of industrial usage of bi- or trimetallic Au catalysts are limited, as these additives do not fully address, but only postpone, the deactivation via reduction and agglomeration and substantially increase the recovery costs of Au from spent catalysts [2, 5, 7, 8].

To stabilize the oxidation state of cationic Au species, non-metal containing additives can be used. Strong heteroatom containing ligands (e.g. sulphate, thiosulphate, trifluorocyanuric acid) have been shown to significantly increase the activity and stability of Au catalysts via enhanced dispersion and stabilisation of cationic Au species against reduction and agglomeration, without significantly increasing the recycling costs of spent catalysts [6, 8, 12]. A thiosulphate ligand stabilized Au catalyst has recently been commercialised by Johnson Matthey [13]. However, preparing these Au^3+^ ligand complexes for impregnation increases the cost, which motivates research on easier alternatives to disperse cationic Au species and stabilise the oxidation state. Further developments are also required to achieve more active Au catalysts which can operate at lower reaction temperatures used in existing reactors [8].

Single atom catalysts have been shown to be stable against reduction and agglomeration by supporting them on carbons with heteroatom containing anchor functionalities on the surface [14–18]. Several studies have independently demonstrated that cationic Au species during acetylene hydrochlorination can be stabilised by the introduction of electron-rich heteroatom functionalities on the surface of the carbon support [2, 3, 6, 7]. However, not all heteroatoms are suitable. Pattisson et al. observed an increased catalytic activity and lower light-off temperature by introducing O containing functionalities on the carbon surface via oxidative treatment with sulphuric and nitric acid (Hummers method), but deactivation via reduction of Au was increased rather than suppressed [8]. Zhao and coworkers reported significant increase in stability of cationic Au species when previously thermally functionalising the activated carbon support surface with urea. They attributed the enhanced catalytic activity and stability to the presence of pyrrole anchor sites that strongly coordinate cationic Au species and donate electron density which stabilised the oxidation state and strengthened the adsorption of HCl and enhanced the activation of C_2_H_2_ [3]. The same group later reported improved catalytic performance of Au supported on activated carbon co-doped with N and S via a treatment with thiourea compared to a non-doped activated carbon support [6]. However, the individual effects of N and S doping were not assessed separately, leaving their respective roles in catalyst stabilisation and activity unclear. Here, we investigate the separate and combined effects of N and S doping on Au catalysts supported on both hard-templated carbon spheres and commercially available activated carbon. The carbon spheres served as model supports, enabling precise control over textural properties so that only the dopant chemistry was varied, thereby allowing direct assessment of the effect of N, S, and N, S co-doping. While these model systems provided valuable comparative insight, their multi-step synthesis and low yields at laboratory scale motivated us to extend the study to an activated carbon doped using a simple gas-phase treatment. In this case, sulphur doping delivered a notable enhancement in VCM production, highlighting the practical potential of S-doped activated carbons as industrially relevant supports. The Au reaction environment is discussed with respect to characterisation and testing data through comparison of doped and undoped catalysts.

## Experimental

### Materials

The following materials were purchased and used without further purification. Extra-dry acetone (Thermo Scientific Chemicals, 99.8% AcroSeal), chloroauric acid (HAuCl_4_.3H_2_O, Alfa Aesar, 99.9%, 49% assay), activated carbon (NORIT ROX 0.8), acetylene/Ar (BOC, 5% balanced in Ar), HCl/Ar (BOC, 5% balanced in Ar), Ar (BOC, N6.0, 99.9999%), ethanol (EtOH, 99.8%), octadecyltrimethoxysilane (OTMS, ≥ 85%) tetraethoxysilane (TEOS, 98%), ammonium hydroxide (25 wt%) solution, nitric acid (HNO_3_, 65%), pyrrole (≥ 98%), thiophene (≥ 99%), thiazole (≥ 99%), HF (40%), ferrocene (> 98%) and sulphur (≥ 99%).

### Doped Carbon Sphere Preparation

Heteroatom-doped carbon spheres were synthesized by chemical vapor deposition (CVD) of heteroaromatic precursors, whereby carbon was deposited within the pores of mesoporous silica serving as a hard template. The mesoporous silica spheres were obtained via the established process reported by Büchel et al. [19]. Building on our previous procedure for hollow graphitic spheres [20], the current synthesis was adapted to employ fully mesoporous (not core shell) silica templates, yielding spherical carbon replicas with uniform mesoporosity, rather than hollow structure. A further adjustment was the use of metal-free heteroaromatic precursors in place of ferrocene, thereby eliminating the catalytic graphitization.

The CVD experiments were performed in a horizontal tube furnace. Mesoporous silica spheres were placed in a quartz crucible at the furnace centre, while the precursor was positioned upstream in a separate chamber equipped with a heating jacket. To minimize undesired precursor evaporation by radiative heating from the furnace, the two chambers were spaced approximately 30 cm apart. A cooling trap was installed downstream of the furnace to capture unreacted vapours and by-products.

The furnace was heated under a flow of high-purity argon (100 mL min^− 1^) at a ramp rate of 10 °C min^− 1^ to 750 °C. Once the furnace reached 50 °C below the target dwelling temperature, the heating jacket was adjusted to 20 °C above the boiling point of the respective precursor to ensure controlled vapor delivery. The vapor infiltration was maintained for 90 min, after which both the furnace and heating jacket were switched off and allowed to cool naturally to room temperature.

The resulting composites were subsequently annealed at 850 °C for 4 h under argon, followed by template removal in aqueous hydrofluoric acid (40 wt%, 7 mL per gram of composite) at room temperature. The products were thoroughly washed with deionised water and ethanol until neutral pH was reached, and dried overnight at 80 °C.

Different heteroaromatic precursors were employed to control the dopant nature within the carbon framework. Thiophene acted as a source of sulphur functionalities, thiazole introduced both nitrogen and sulphur species, and pyrrole contributed predominantly pyrrolic nitrogen. In all cases, the heteroaromatic molecules simultaneously served as carbon sources, enabling uniform incorporation of the desired dopants while preserving the spherical mesoporous architecture directed by the silica hard template. The resultant carbons are referred to as **sC**, **nC** and **nsC**, where the s or n refer to sulphur and/or nitrogen doping of the carbon sphere.

For comparison, a reference carbon sphere without heteroatom doping was synthesized using the same mesoporous silica template and CVD setup. In this case, ferrocene was employed as the carbon precursor. As reported previously [20], Fe can catalyse graphitization; therefore, the CVD composites were annealed at a lower temperature of 800 °C for 4 h under argon to suppress graphitization and obtain undoped carbon spheres with a low degree of graphitisation comparable to the doped samples. After annealing, the silica template was removed using aqueous hydrofluoric acid (40 wt%, 7 mL per gram of composite) at room temperature, following the same procedure as for the doped materials. The products were thoroughly washed with deionized water and ethanol until neutral pH was reached, then leached with half-concentrated HCl at 60 °C overnight to remove residual Fe, washed again, and finally dried at 80 °C overnight. The reference carbon is hereafter referred to as **rC**.

### Doped Activated Carbon Preparation

The post-functionalisation of commercial activated carbon (Norit; denoted as **C**) was adapted from procedures reported in the literature [21]. In a typical synthesis, 7 g of Norit carbon were double sieved between 90 and 250 μm, then oxidized in 400 mL of concentrated nitric acid (70 wt%) under reflux conditions at 70 °C for 30 min with continuous stirring. After cooling, the mixture was carefully diluted with water, followed by filtration and through washing with water and ethanol. The oxidized carbon was then dried in air at 75 °C and named as **O-Norit**.

#### Nitrogen Doping

The pre-oxidized Norit carbon was subjected to ammonia treatment in a tube furnace under a constant flow of NH_3_ (100 mL min^− 1^). Two different conditions were applied: either 4 h at 400 °C or 1 h at 700 °C, in both cases using a heating rate of 10 °C min^− 1^. After the thermal treatment, the furnace was allowed to cool down naturally to room temperature and purged with argon for 1 h. The sample was subsequently washed with water and ethanol and dried in air at 75 °C hereafter referred to as **N-400**, and **N-700**, respectively.

#### Sulphur Doping

For sulphur functionalization, 500 mg of pre-oxidized Norit carbon and 2 g of elemental sulphur were placed in separate crucibles inside a tube furnace under an argon flow of 100 mL min^− 1^. The furnace was heated to 300 °C at a rate of 5 °C min^− 1^ and maintained for 1 h, followed by a second step at 500 °C for 3 h to remove excess sulphur species. A washing bottle was connected to the exhaust line of the tube furnace as a safety precaution to capture volatile sulphur compounds. After cooling to room temperature, the resulting S doped Norit (denoted as **S-Norit**) was washed with toluene and dried under vacuum at 100 °C.

### Catalyst Preparation

A 1.0 wt% Au/C catalyst was prepared according to the following procedure [22]. HAuCl_4_.3H_2_O (Alfa Aesar, 20 mg, assay 49%), was dissolved in dry acetone (2.7 g) and the solution was stirred for 10 min. The gold solution was added dropwise while stirring to activated carbon (powdered NORIT ROX 0.8; sieved to 90–150 μm, 0.99 g) to achieve a metal loading of 1.0 wt%. The catalyst was dried under nitrogen flow at 45 °C for 2 h and denoted as **Au/C**. The same procedure was followed for the other carbons prepared in this study to form 1 wt% Au catalysts for comparison to the Au/C standard.

### Catalyst Testing

Unless otherwise stated, all reactions were conducted using the following conditions. The reactor was purged with Ar (99.99%, Air products) prior to introduction of reactant gases. Typically, the reactor was heated to 180 °C for 30 min while under constant flow of Ar (50 mL min^− 1^). C_2_H_2_ (5.01% in Ar, BOC, 23.56 mL min^− 1^), HCl (5.05% in Ar, BOC, 23.76 mL min^− 1^) and Ar (2.70 mL min^− 1^) were then introduced to the reactor which contained catalyst (90 mg) giving a total flow of 50 mL min^− 1^ (~ 17,600 h^− 1^ GHSV) and a C_2_H_2_:HCl ratio of 1:1.02 under ambient pressure. Full conversion and selectivity under these conditions would provide a VCM productivity of 35.33 mol kg_cat_^−1^ h^− 1^. Analysis of the acetylene hydrochlorination reaction was carried out using a Varian CP-3800 GC fitted with a Poropak-N packed column and an FID detector. Conversion of acetylene was calculated using Eq. 1, where *APa*_*i*_ and *APa*_*f*_ are the initial and final GC peak areas of acetylene respectively. In all cases, only VCM was detected as product, which is in agreement with previous reports on Au catalysts prepared using this technique [22].

1 $${\mathrm{Acetylene~conversion}}~\left( \% \right)=\frac{{AP{a_i} - AP{a_f}}}{{AP{a_i}}} \times 100$$

### Characterisation

Powder X-ray diffraction (XRD) patterns of the carbon supports were recorded on a STOE STADI P diffractometer operating in Bragg–Brentano geometry with Cu Kα radiation (λ = 1.5418 Å). A secondary graphite monochromator was used, and samples were mounted on a background-free holder. The divergence and receiving slits were set to 0.8° and 0.8 mm, respectively. Powder X-ray diffraction (XRD) analysis of supported catalysts was performed between 10° and 80° 2θ using an X’Pert Pro PAN Analytical powder diffractometer employing a Cu Kα radiation source operating at 40 keV and 40 mA. Analysis of the spectra obtained was carried out using X’Pert High Score Plus software.

Thermogravimetric analysis (TGA) of the carbon supports was performed using a Netzsch STA 449 F3 Jupiter thermal analyser. Approximately 5 mg of sample was heated from 45 to 900 °C at a rate of 10 °C min^− 1^ under a continuous flow of oxygen (40 mL min^− 1^). In all measurements, an additional protective argon flow of 20 mL min^− 1^ was applied.

Transmission electron microscopy (TEM) micrographs of the carbon supports were obtained using a Hitachi H-7500 microscope (100 kV, tungsten filament). All samples were deposited on copper grids coated with a lacey carbon film.

Field Emission Gun - Scanning Electron Microscopy (FEG-SEM) was performed using a Tescan Maia3 operating at 3 kV. Sample were dry dispersed onto carbon Leit discs, mounted on 12.5 mm aluminium stubs and imaged uncoated.

X-ray photoelectron spectroscopy (XPS) measurements of the carbon supports were collected using a SPECS GmbH instrument equipped with a PHOIBOS 150 hemispherical energy analyzer and a 1D delay line detector (DLD). A monochromatized Al Kα X-ray source (E = 1486.6 eV) was operated at 15 kV and 200 W. To compensate for the positive charging effects of insulating sample surfaces, an electron flood gun (FG500X, SPECS GmbH) was operated at 400 V and 200 µA. Survey scans were acquired using a pass energy of 50 eV, while high-resolution narrow scans were recorded with a pass energy of 20 eV. The vacuum inside the analysis chamber was maintained at 5·10^− 10^ mbar during all measurements. Data processing was carried out using the CasaXPS (Version 2.3.25) software package [23], and all spectra were corrected for charging by referencing the C 1s signal at 284.5 eV. XPS characterization was performed by Sebastian Leiting from the group of Prof. Claudia Weidenthaler.

Nitrogen physisorption measurements were conducted using a Micromeritics 3Flex surface characterization analyser. Prior to the measurements, the samples were degassed under vacuum at 300 °C for 12 h to ensure proper activation. The adsorption analyses were carried out at 77.4 K using liquid nitrogen as the coolant. The specific surface area was determined using the Brunauer–Emmett–Teller (BET) method, based on the desorption isotherm within the relative pressure range of 0.05 to 0.25 p/p_0_. The total pore volume was estimated at a relative pressure of 0.9 p/p_0_ using the Gurvich rule. Pore size distribution was obtained by applying the Density Functional Theory (DFT) model, assuming slit-shaped carbon pores.

## Results and Discussion

### CVD of Heterocycles towards Doped Carbons Spheres

To enhance the anchoring of Au, uniformly doped carbon spheres were synthesized via chemical vapour deposition (CVD) of heteroatom-containing precursors [20]. This approach was selected as it typically enables a higher degree of heteroatom incorporation while ensuring homogeneous doping throughout the carbon matrix. To preserve comparable textural properties, a hard-templating method was employed, which maintains a similar morphology and porosity when different precursors are used. As a result, the obtained carbon materials exhibit a high heteroatom content, nearly identical morphology, and comparable degrees of graphitization, making them ideal model systems. Pyrrole, thiophene, and thiazole were chosen as C and heteroatom sources due to their rich N and/or S content, low toxicity, favourable volatility under CVD conditions, and controlled decomposition within the pores of the silica template. For comparison, undoped carbon spheres were synthesized using ferrocene as the carbon source. To minimize Fe-catalysed graphitization, which would otherwise alter the structural characteristics, a slightly lower annealing temperature was applied, and the residual Fe was removed during post-treatment [20]. As a result, the reference carbon spheres displayed morphology and textural properties closely resembling to those of the doped materials, enabling a clear evaluation of the effect of heteroatom incorporation on the performance.

**Fig. 1:**
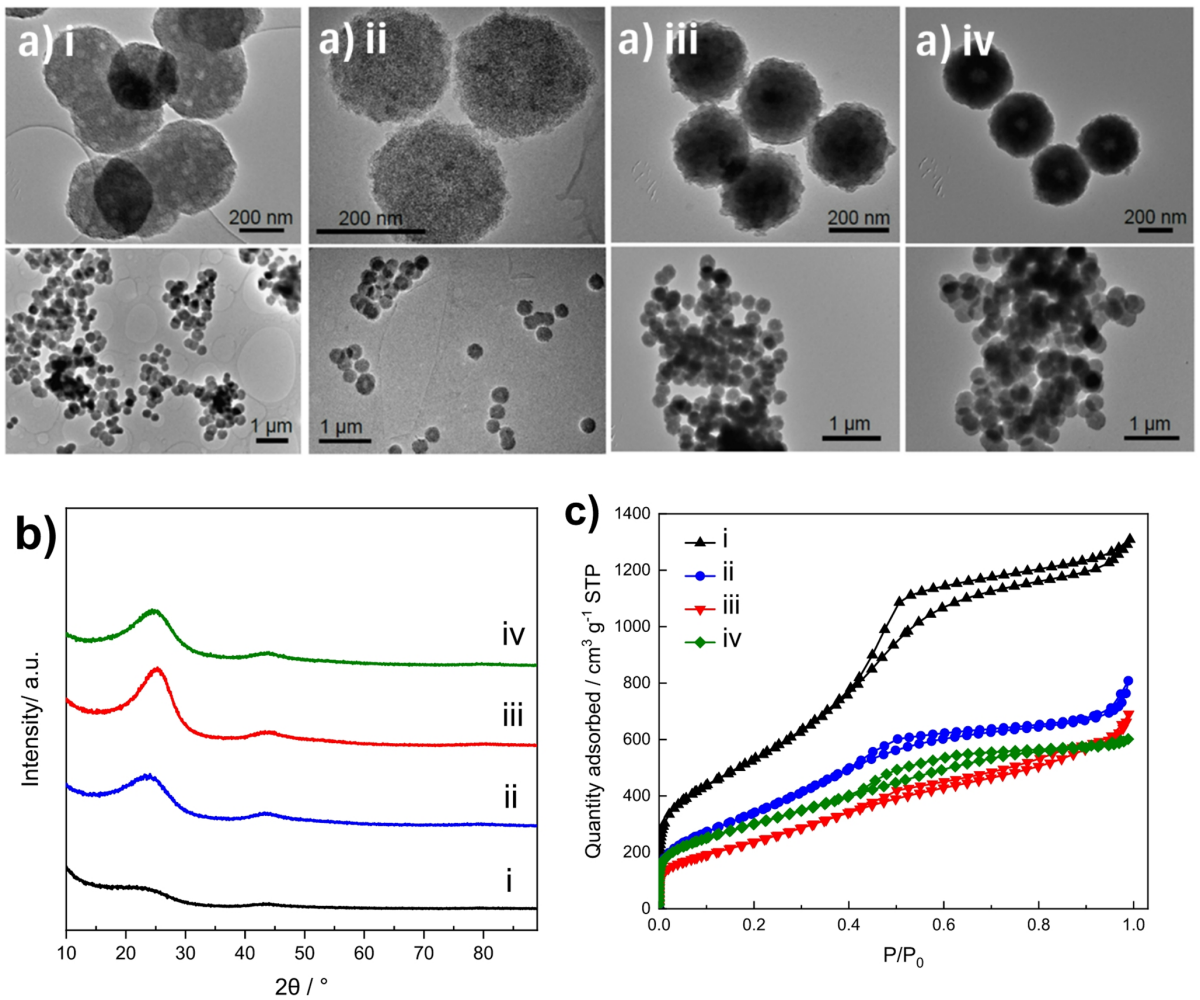
Characterisation of the X-Doped carbon spheres; **a** representative TEM images; **b** powder XRD patterns and **c** N_2_ adsorption-desorption isotherms. (i) rC, (ii) sC, (iii) nC and (iv) nsC

As prepared doped carbon spheres were characterised with TEM, XRD, N_2_ physisorption (Fig. 1), and TGA (Fig. S1). Well-defined, uniform carbon spheres were observed in the TEM images (Fig. 1a), showing a consistent diameter of ~ 300 nm independent of the dopant used. The XRD patterns exhibit broad graphite reflections at ~ 25° and ~ 43°, characteristic of an amorphous structure with a low degree of graphitization, similar to conventional carbon blacks (Fig. 1b). Subtle shifts in the position and intensity of the (002) reflection were further observed across the doped samples, in line with literature reports on heteroatom-doped carbons where incorporation of N or S modifies graphitic ordering and interlayer spacing [24, 25].

The textural properties of the carbon spheres were evaluated through N_2_ physisorption. All samples exhibit type IVa isotherms according to IUPAC classification [26], characteristic of materials with small mesopores. The isotherms show a similar slope at low relative pressures (0–0.4 P/P_0_), corresponding to an average pore size of ~ 3–4 nm. The characteristic bend at ~ 0.4 P/P_0_ corresponds to mesopore filling and is accompanied by a type H3 hysteresis loop. The reference material displays enhanced mesoporosity, evidenced by a pronounced nitrogen uptake at high relative pressures and a larger hysteresis loop, likely resulting from the slightly modified synthesis route (Fig. 1c). Since the BJH method underestimates narrow mesopores (< 10 nm) by up to ~ 20–30% [26], pore size distributions were calculated using NLDFT with slit-pore geometry, providing a more accurate description of the carbon materials’ porosity (Fig. S2) [27]. Consequently, the specific surface areas of the carbon spheres range from 1971 m^2^ g^− 1^ of rC to 1308 m^2^ g^− 1^ (sC), 907 m^2^ g^− 1^ (nC) and 1089 m^2^ g^− 1^ (nsC) (Table S1). The respective pore volumes were measured to be 1.95, 1.10, 0.94 and 0.91 cm^3^ g^− 1^.

### Spherical Carbon Supported Catalysts

**Fig. 2:**
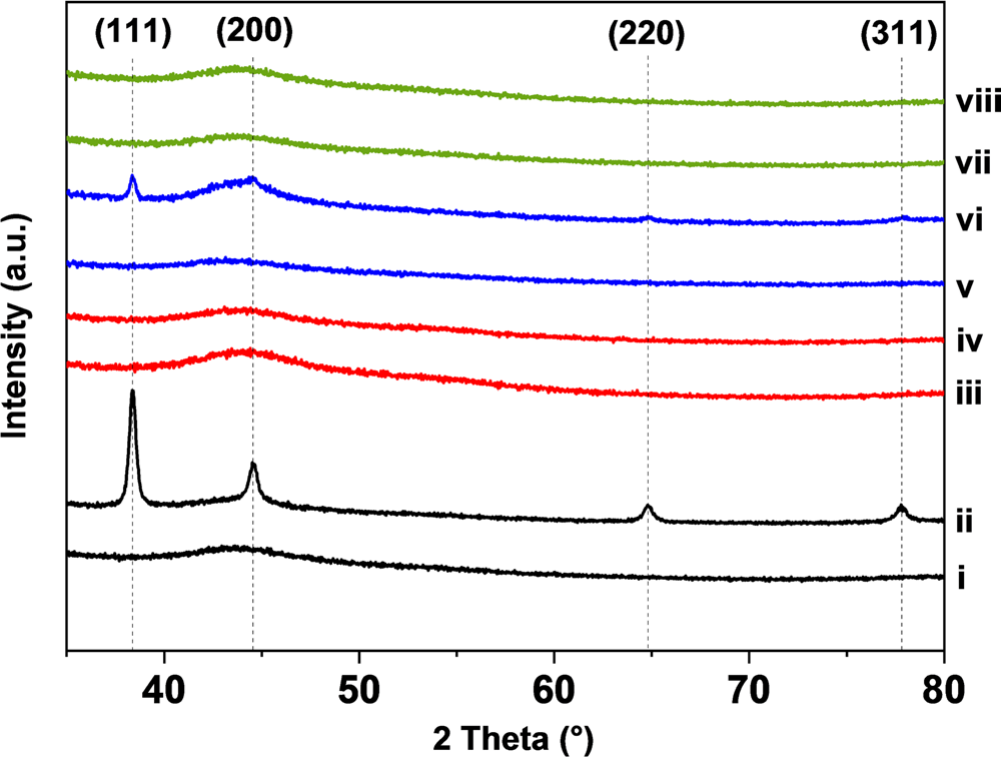
Powder XRD of the X-doped carbon sphere catalysts (1% Au/*x*C), as prepared and post-reaction; (i) rC, (ii) rC-tested, (iii) nC, (iv) nC-tested, (v) sC, (vi) sC-tested, (vii) nsC, (viii) nsC-tested. Dashed lines correspond to *hkl* reflections of Au nanoparticles

**Fig. 3:**
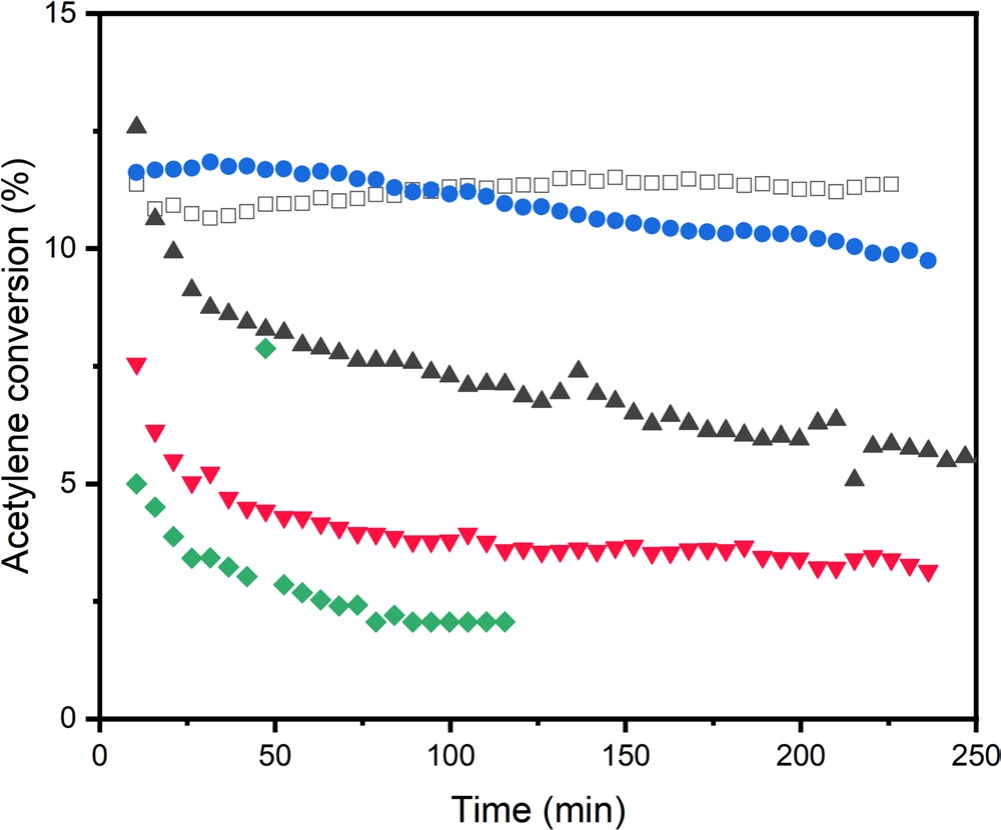
Acetylene conversion during hydrochlorination reaction over doped and undoped sphere catalysts compared to standard Norit catalyst 1%Au/C (open black square). 1%Au/rC (black filled triangle); 1%Au/sC (violet filled circle); 1%Au/nC (red inverse filled triangle); 1%Au/nsC (green filled diamond). Reaction conditions; 45 mg of sample, 11.10 mL min^− 1^ C_2_H_2_ (5% acetylene in argon), 11.75 mL min^− 1^ HCl (5% hydrogen chloride in argon) and 2.15 mL min^− 1^ Ar, 180 °C

Au was added through an established procedure with dry acetone and the catalysts were then used to react HCl and acetylene under dilute conditions [22]. The catalysts were initially examined by XRD prior to use, which revealed no significant nanoparticle presence above the detection limits of the technique (Fig. 2). This is in agreement with our previous work which demonstrated the ability of this preparation method to produce atomically dispersed supported gold catalysts [22]. Figure 3 illustrates the reactivity profiles of the catalysts as a function of time. The 1%Au/C catalyst prepared on activated carbon was stable across the 4 h reaction, with an acetylene conversion of *ca*. 13%. In contrast, the 1%Au/sC initially was slightly more active within the first hour time-on-line, however, activity decreased from 12 to 10% over 4 h. Dawson et al. reported enhanced acetylene conversion with sulphur-modified Au/C catalysts and noted that following a significant induction period to high activity with H_2_SO_4_ washed catalysts, a steady deactivation was observed [12]. They related the activity enhancement to reduction in Au mobility when sulphur was present. Similarly, Song et al. observed a reduced deactivation rate with sulphur treated Au catalysts in comparison to the untreated spherical activated carbon analogue [28]. The formation of Au nanoparticles under reaction conditions is commensurate with deactivation [29]. In contrast, the initial high activity of 13% over the 1%Au/rC catalyst significantly decreased over 4 h, and only 6% conversion was achieved at 4 h. Both N- and S, N-doped catalysts were poorly active, with conversions of < 5% over the 4 h. However, a comparable trend of initially higher relative activity can be seen for the rC, nC and nsC-based catalysts which suggests that the Au speciation was not stable over the reaction time [29, 30].

The significant deactivation observed with the doped carbon sphere catalysts, with the exception of 1%Au/sC, was explored with XRD to see if Au sintering had occurred. Au catalysts are known to deactivate over long reaction times via reduction and agglomeration to inactive Au nanoparticles [29]. Diffraction patterns of the as-prepared and used catalysts, after 4 h are illustrated in Fig. 2. Clear sharp reflections are visible from XRD measurements of the 1%Au/rC sample, which correspond to Au nanoparticles with an average crystallite size of 35 nm (Fig. 2). Their presence supports the deactivation profile observed in Fig. 3. In contrast, the low activity of the nC and nsC-based catalysts cannot be related to the formation of Au nanoparticles > 4 nm as no Au reflections are present in the XRD to support this inference. However, reflections are observable in the XRD pattern of the used 1%Au/sC catalyst sample albeit of low intensity. The modest deactivation observed over 4 h TOL supports this and suggests that the S-doping can stabilise the reactive Au centres when compared to the undoped carbon spheres (1%Au/rC). However, this effect is not long lasting and, in comparison to the 1%Au/C, there is no enhancement in VCM productivity.

**Fig. 4:**
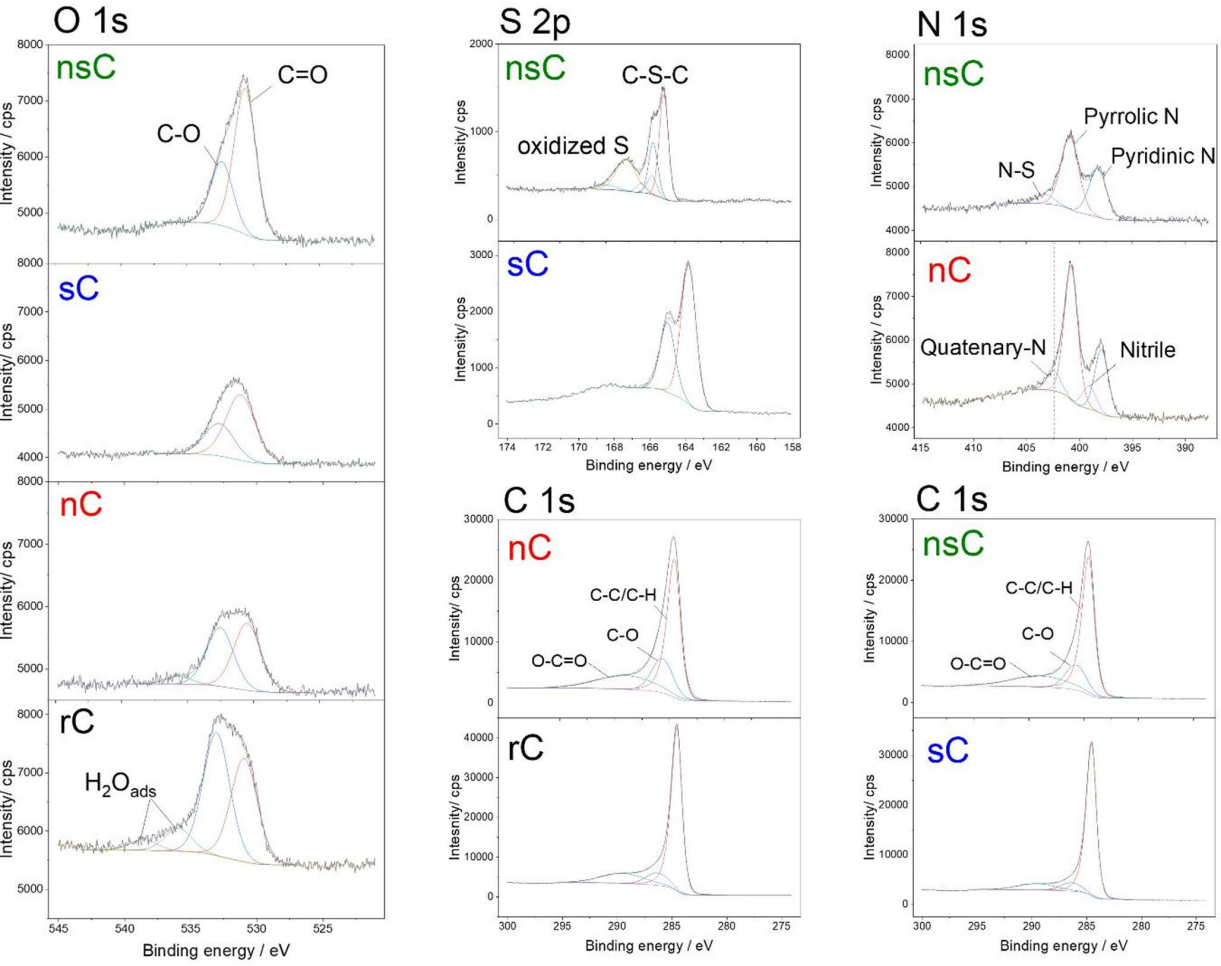
Deconvoluted XPS O 1s, S 2p, N 1s, and C 1s spectra of the doped carbon spheres prepared using different heterocycles as precursor

The differences in performance can be rationalised by considering the surface chemistry revealed by XPS (Fig. 4 and Table S2). The nsC sample contained a high proportion of carbonyl oxygen (70% of O1s) along with a substantial fraction of oxidised sulphur species (binding energies > 167 eV); with 4.0 at% O, 4.0 at% S and 5.6 at% N. Such oxidised species are known to impart acidity and oxidative character, which may hinder Au stabilisation and promote unfavourable side reactions, accounting for the very low initial activity and poor overall performance. In contrast, the sC contained almost exclusively low-valence thiophenic sulphur (S2p peaks at 163–165 eV, 6.2 at% S), and no oxidised S, correlating with its higher activity. These findings support the view that specific sulphur functionalities can act as soft donors to stabilise cationic Au, as reported by Hutchings and co-workers, who showed that thiosulfate ligands prevent sintering of Au(I) and sustain activity in acetylene hydrochlorination [13]. Dawson et al. also demonstrated promotion following sulphur functionalisation of carbon supports [12], although the precise identity of the active sulphur species was not resolved. Duan et al. provided a broader perspective across different metals, showing that high-valence sulphur groups such as –SO₃H stabilised Au cations and improved stability, whereas low-valence –SH favoured reduction to Au^0^ and deactivation [31]. Our observations, however, point to the opposite trend, where oxidised sulphur correlates with poor performance and thiophenic sulphur with higher activity. This discrepancy highlights the importance of the specific carbon environment and possible interactions with co-dopants. In support of this, Xiao-Xia Di et al. reported that N, S co-doped carbons with a relatively small fraction of oxidised sulphur species (approximately 14%) and higher proportion of thiophenic sulphur, stabilised Au^3+^ and enhanced both activity and stability [6]. Their results indicate that N and S heteroatoms can inhibit the reduction of Au^3+^ to Au^0^ under reaction conditions, thereby improving long-term performance [6].

The sC sphere, with a balanced O 1s distribution between carbonyl (67%) and C–O (33%) groups, showed the highest activity, whereas the nsC sphere, dominated by carbonyl oxygen, and the rC sphere, richer in C–O, both performed poorly. Prior studies on Pt SACs supported on carbon have shown that abundant acidic oxygen groups promote coke formation and reduce stability [32]. Our findings indicate a similar effect for Au: while moderate oxygen functionalities help disperse and anchor Au species, excessive acidic or oxidising groups hinder durability.

The nC sphere contained a high fraction of pyrrolic N (∼56%), alongside pyridinic, quaternary, and nitrile species with a total N at% of 7.4; and exhibited markedly lower catalytic performance than the sC sphere or standard Au/Norit. This highlights the importance of N speciation: pyridinic N can stabilise Au species, whereas pyrrolic N has been linked to enhanced coking in metal-supported catalysts, as demonstrated in computational studies on Pt and Ru [33]. For non-metal-based catalysts [34], however, the effects of nitrogen functionalities may differ, reflecting the complex but distinct interactions between cationic gold species, heteroatom dopants and the support.

The C 1s spectra is deconvoluted to the main reference peak at 284.5 eV corresponding to alkyl type carbon (C-C/C-H), and the peak around 285.5–286.3 eV representing C-O. The higher binding energies around 290 eV is related to the other oxygenated compounds such as O-C = O, but can also be overlap of C = O and O-C = O.

The modest enhancement in VCM productivity over the S-doped carbon sphere catalyst in comparison to the rC-based catalyst supports previous reports [12] that heteroatom doping is a viable catalyst design methodology. Consequently, the study was expanded to include doping commercial microporous, activated carbon with O, N and S via reaction with HNO_3_, NH_3_ and sulphur respectively, as set out in the Experimental section. As this AC can be commercially sourced, this would remove the need for the challenging preparation of the carbon spheres but retain the potential for Au stabilisation as discussed above and provide a more industrially relevant study.

The modified Norit carbons were characterised by N_2_ physisorption (Fig. S3), XPS (Fig. S4), and SEM (Fig. S5). As expected, the chemical post-treatments increased the surface heteroatom contents relative to the parent carbon and decreased the specific surface area (Table S1). For instance, nitric acid oxidation increased the O content from *ca*. 4 to 8 at%, sulphur treatment introduced *ca*. 2 at% S, and NH_3_ treatment yielded ca. 3 at% N at 400 °C and ca. 2 at% at 700 °C (Table S2 and Fig. S4). These changes were accompanied by shifts in speciation, with oxidised Norit displaying higher shares of C–O groups, while s-doped Norit retained a balanced C = O/C–O distribution from XPS analysis (Fig. S4). The specific surface area of the parent Norit carbon was calculated with the BET equation to be 1446 m^2^ g^-1^, and when modified decreased to 1426, 1260, 1360 and 1327 m^2^ g^-1^ respectively for O-Norit, S-Norit, N400 and N700. The pore volume of the modified carbons remained comparable to the parent carbon at ca. 0.78 cm^3^ g^-1^, only the S-Norit was significantly different at 0.68 cm^3^ g^-1^.

**Fig. 5:**
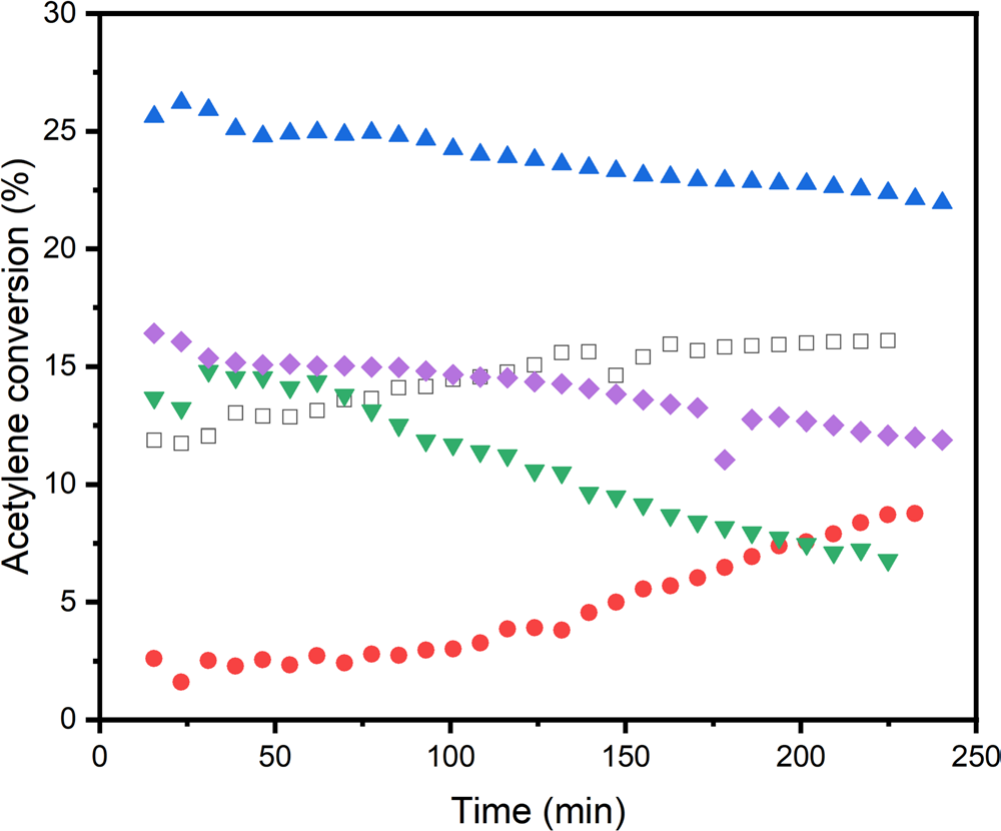
Hydrochlorination of acetylene testing over 1wt.% Au/X-Doped Norit catalysts. C (black open square); O-Norit (red filled circle); S-Norit (violet filled triangle); N700 (green inverse filled triangle); N400 (purple filled diamond). Reaction conditions; 90 mg of sample, 22.95 mL min^− 1^ C_2_H_2_ (5% acetylene in argon), 23.50 mL min^− 1^ HCl (5% hydrogen chloride in argon) and 3.55 mL min^− 1^ Ar, 180 °C

Catalytic testing revealed pronounced differences, as illustrated in Fig. 5. O-Norit was initially almost inactive, only gradually gaining activity over several hours on stream. Previously, we have shown [8] that by refluxing AC in nitric acid resulted in a relatively long induction period over the subsequently prepared aqua-regia deposited Au catalyst, which is in line with the findings presented here. In contrast, S-Norit exhibited a striking enhancement in activity, surpassing the performance of the standard 1%Au/C, however, the catalyst stability was relatively poor, and a modest deactivation rate can be observed. Here the surface chemistry was dominated by thiophenic S with negligible oxidised S (*ca*. 4.6%), reinforcing the conclusion that oxidised S species may be detrimental for activity in this system. In situ XAS experiments would be required to confirm whether differences in performance are due to any modification of Au oxidation resulting from the modified supports. Analysis of the post-use SEM images (Fig. S5) suggests that Au sintering has occurred with S-Norit and is likely the reason for the deactivation, albeit at only a modest rate. N-Norit showed similar activity to the parent Norit catalyst, consistent with the predominance of pyridinic N stabilising Au without additional promotion of acetylene activation. Both N-doped Norit catalysts deactivated with time on stream, but the N700-Norit sample which was treated at 700 °C performed particularly poorly. The higher proportion of pyrrolic nitrogen (40% compared with 22% in the N400-Norit sample) in that sample is consistent with earlier findings that pyrrolic N can accelerate coking and catalyst deactivation.

## Conclusion

Overall, these findings emphasise that the decisive factors contributing to improved catalytic performance of Au/C in acetylene hydrochlorination are not merely the presence of heteroatoms, but also their specific chemical state, spatial distribution and arrangement, as well as the porous architecture of the support. The comparison between mesoporous templated spheres and microporous Norit suggests that microporosity may promote stronger acetylene adsorption and local enrichment near active sites. Sulphur incorporation in a microporous carbon framework appears especially effective for balancing Au stabilisation and acetylene interaction, whereas excessive O functionality hinders both activity and stability. N doping can stabilise Au single atoms when pyridinic N is dominant, but high pyrrolic fractions correlate with weaker activity and faster deactivation. These insights highlight the importance of tailoring both the type and chemical state of heteroatoms, together with the porosity of the carbon support, for the rational design of stable and active Au catalysts for acetylene hydrochlorination.

## Supplementary Information

Below is the link to the electronic supplementary material.

Supplementary Material 1

## Data Availability

The authors declare that the data supporting the findings of this study are available within the paper and the corresponding Supplementary Information file.

## Abbreviations

AC: Activated carbon
BET: Brunauer–Emmett–Teller
BJH: Barrett-Joyner-Halenda
CAGR: Compound annual growth rate
CVD: Chemical vapor deposition
DFT: Density functional theory
EDC: Ethylene dichloride
NLDFT: Non-local density functional theory
PVC: Polyvinyl chloride
SEM: Scanning electron microscopy
TEM: Transmission electron microscopy
TGA: Thermal gravimetric analysis
VCM: Vinyl chloride monomer
XPS: X-ray photoelectron spectroscopy
XRD: X-ray diffraction
