# Supplementary material for: Cationic Gold on Heteroatom Doped Carbon Supports for Vinyl Chloride Production
Source: Catal Letters. 2026 Mar 18;156(4):104. doi: 10.1007/s10562-026-05351-2 (PMC12999652; doi:10.1007/s10562-026-05351-2)
Supplement: Supplementary file 1 — Supplementary Material 1 [file 10562_2026_5351_MOESM1_ESM.docx]

Supplemental Information

**Cationic gold on heteroatom doped carbon supports for vinyl chloride production.**

Joseph Cartwright^1ψ^, Hannaneh Hosseini^2ψ^, Alexander Gunnarson^2^, Anna Lazaridou^1^, Jonathan M. Mauß^2^, Ben Davies^1^, Samuel Pattisson^1^, Angeles Lopez-Martin^1^, David J. Morgan^1^, Nicholas F. Dummer^1^, Ferdi Schüth^2^, Graham J. Hutchings^1^*

^1^ Max Planck-Cardiff Centre on the Fundamentals of Heterogeneous Catalysis FUNCAT, Cardiff Catalysis Institute, School of Chemistry, Cardiff University, Translational Research Hub, Cardiff, CF24 4HQ, UK.

^2^ Max-Planck-Institut für Kohlenforschung, Department of Heterogeneous Catalysis, Kaiser-Wilhelm-Platz 1, 45470 Mülheim an der Ruhr, Germany.

^ψ^ These authors contributed equally to this study.

* Corresponding author: [schueth@kofo.mpg.de](mailto:schueth@kofo.mpg.de), [Hutch@cardiff.ac.uk](mailto:Hutch@cardiff.ac.uk)

| **Table S1** Textural properties of the carbons used in this study; spheres and doped-Norit. | | |
| --- | --- | --- |
| **Sample** | **Specific surface area^a^**  **/m^2^ g^-1^** | **Pore volume^b^**  **/cm^3^ g^-1^** |
| rC | 1971 | 1.95 |
| sC | 1308 | 1.10 |
| nC | 907 | 0.94 |
| nsC | 1089 | 0.91 |
| C (Norit) | 1446 | 0.78 |
| O-Norit | 1426 | 0.77 |
| S-Norit | 1260 | 0.67 |
| N400-Norit | 1360 | 0.72 |
| N700-Norit | 1327 | 0.71 |
| ^a^ as determined from N_2_ desorption with the BET method; ^b^ as determined with a relative pressure of 0.9 p/p₀ using the Gurvich rule. | | |

| **Table S2** XPS elemental analysis (at.%) of X-Doped carbon spheres and X-doped Norit. | | | | | | | | | |
| --- | --- | --- | --- | --- | --- | --- | --- | --- | --- |
| **Element** | **Carbon sphere** | | | | **Norit** | **X-doped Norit** | | | |
|  | **rC** | **sC** | **nC** | **nsC** | **C** | **O** | **S** | **N400** | **N700** |
| **C** | 95.7 | 90.3 | 90.3 | 86.4 | 95.8 | 91.7 | 93.3 | 93.42 | 94.5 |
| **O** | 3.7 | 3.5 | 2.1 | 4.0 | 4.2 | 8.3 | 2.5 | 3.9 | 3.1 |
| **N** |  |  | 7.4 | 5.6 |  |  |  | 2.6 | 2.3 |
| **S** |  | 6.2 |  | 4.0 |  |  | 3.6 |  |  |

**Fig. S1** TGA of as prepared X-doped carbon spheres under flowing air.

**Fig. S2** Pore size distribution of X-doped carbon spheres as determined with Density Functional Theory (DFT) model, assuming slit-shaped carbon pores.

**Fig. S3** N_2_ adsorption-desorption isotherms of X-doped Norit.


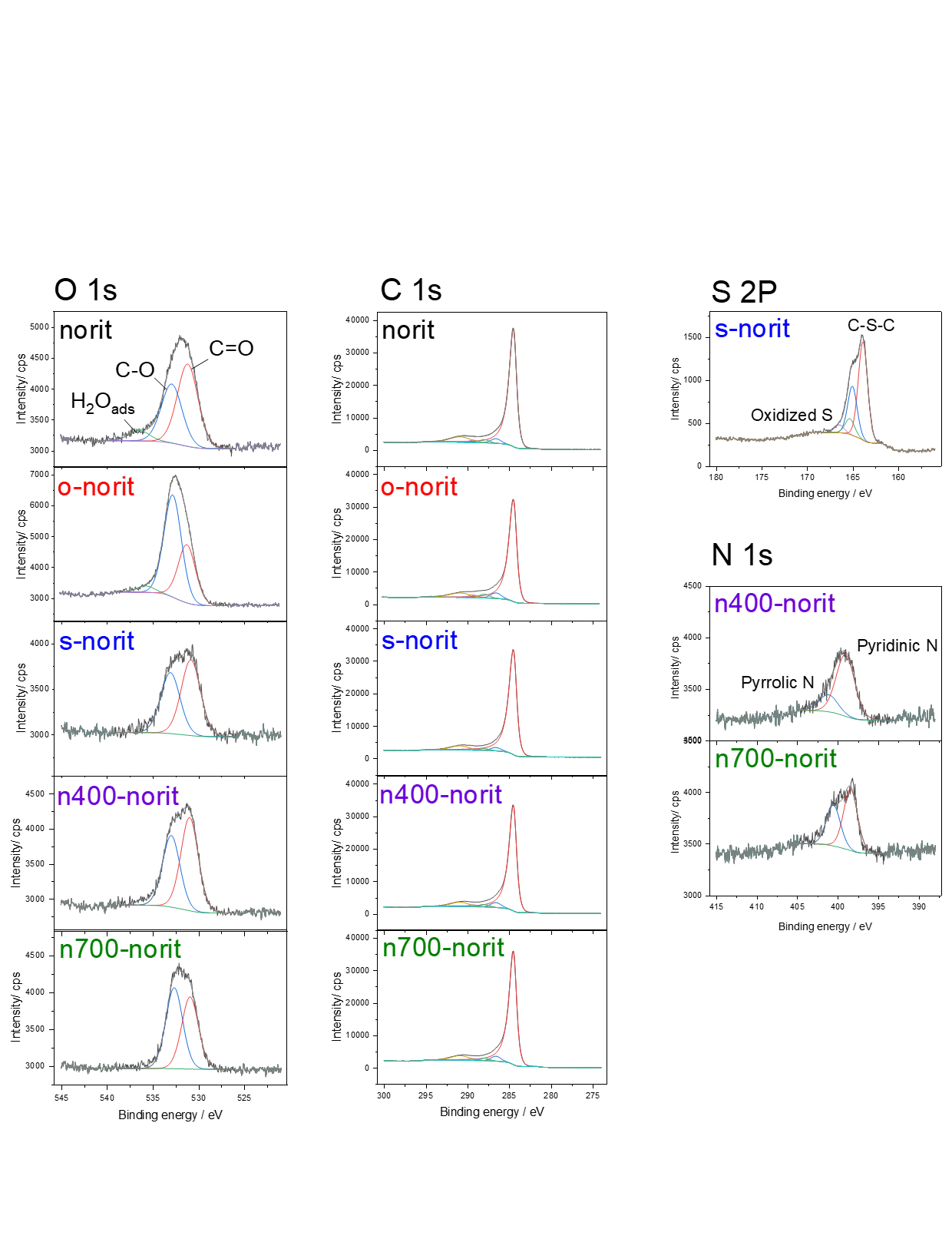


**Fig. S4** Deconvoluted XPS O 1s, C 1s, S 2p, and N 1s spectra of the doped Norit carbon prepared via post treatment of Norit activated carbon.

**
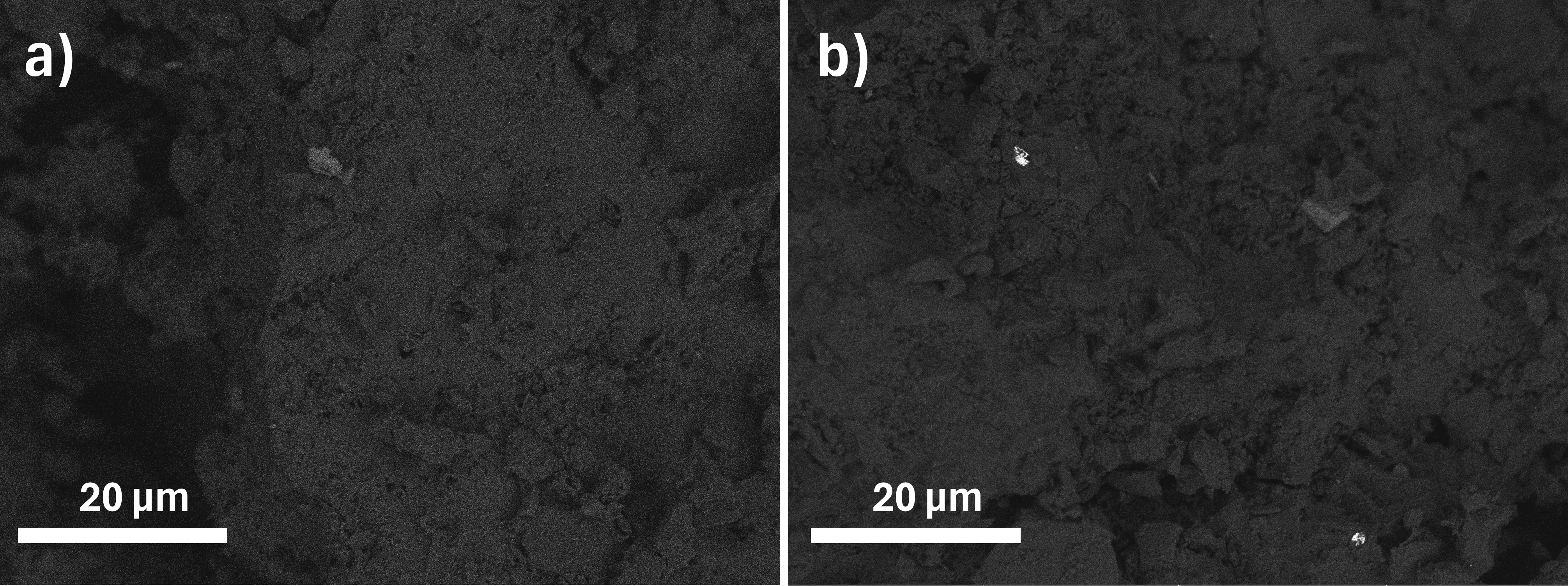
**

**
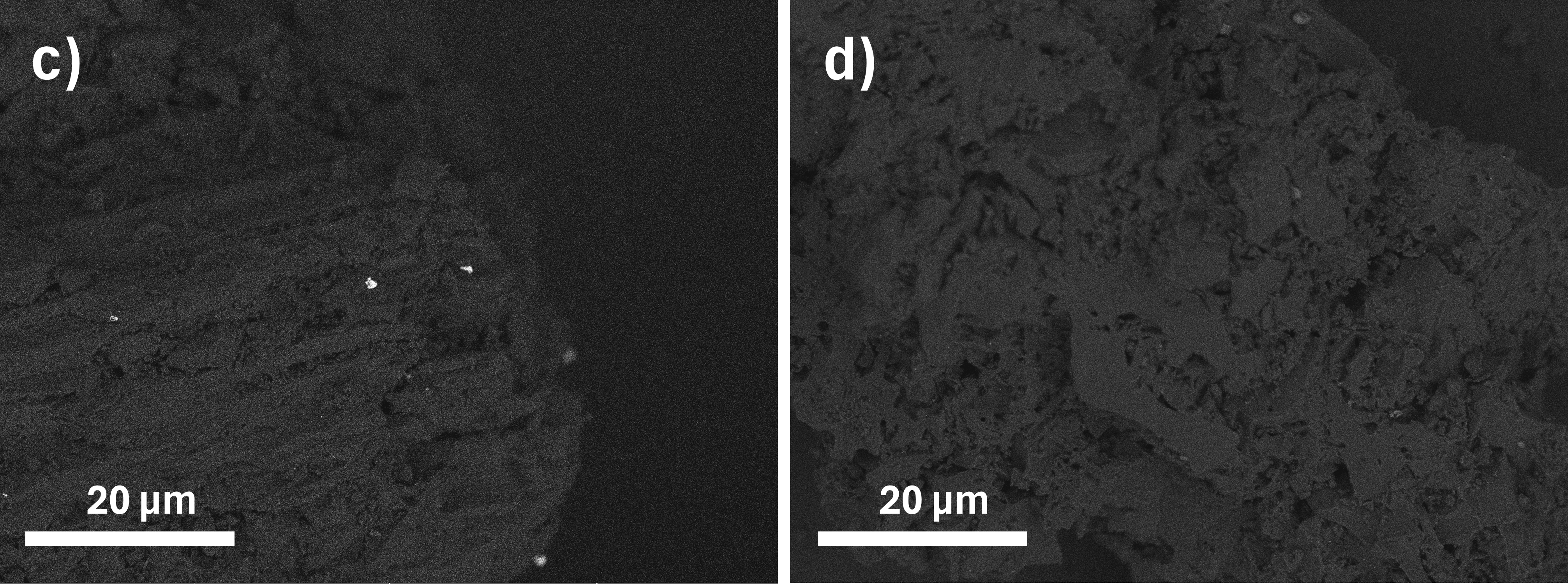
**

**
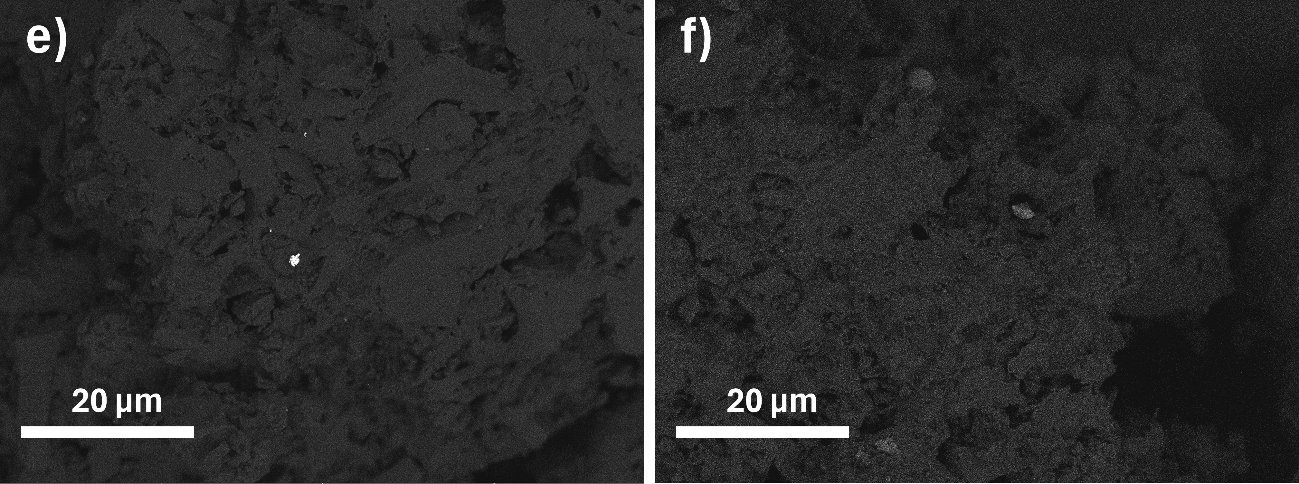
**

**
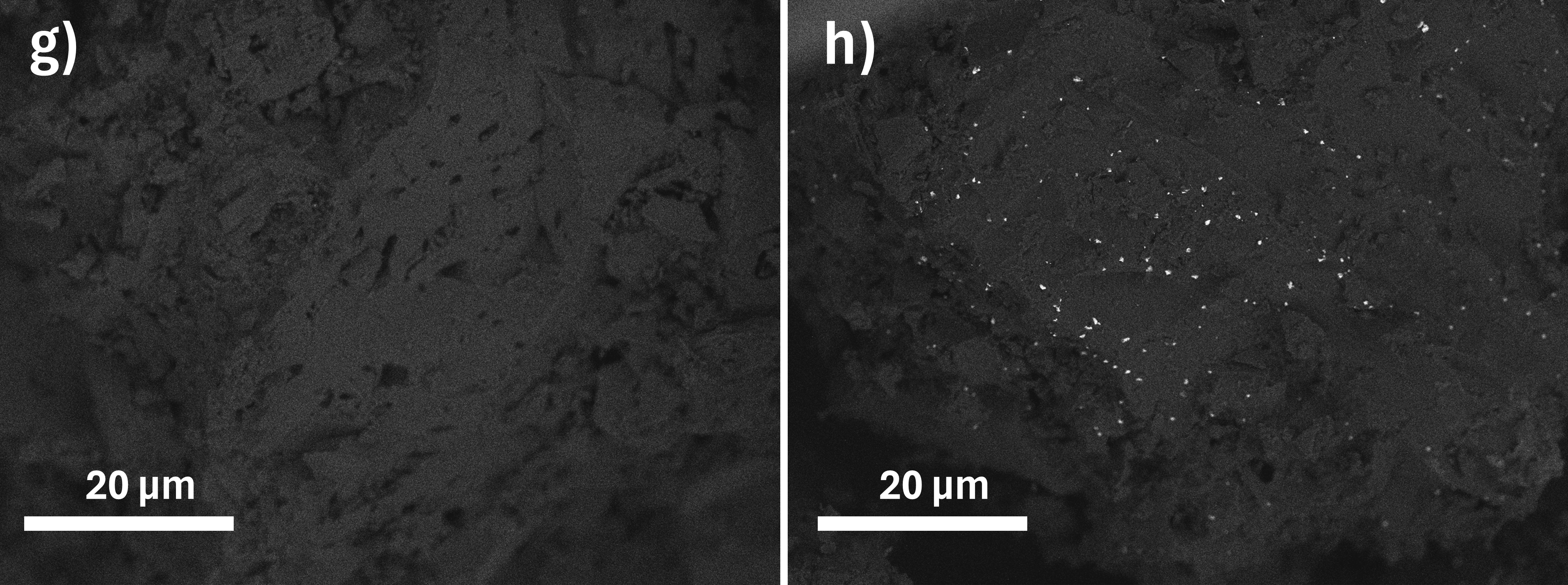
**

**Fig. S5** Representative SEM images of as prepared (left) and used (right) 1%Au/C O-Norit (a and b), 1%Au/C N400-Norit (c and d), 1%Au/C N700-Norit (e and f) and 1%Au/C S-Norit (g and h) modified catalysts.
